# Comparison of risks of cardiovascular events in the elderly using standard survival analysis and multiple-events and recurrent-events methods

**DOI:** 10.1186/s12874-015-0004-3

**Published:** 2015-03-08

**Authors:** Edward H Ip, Achmad Efendi, Geert Molenberghs, Alain G Bertoni

**Affiliations:** Department of Biostatistical Sciences, Wake Forest School of Medicine, Medical Center Boulevard, Winston-Salem, NC 27157 USA; Programs of Statistics, Department of Mathematics, Universitas Brawijaya, Malang, 65145 Indonesia; I-BioStat, Universiteit Hasselt & KU Leuven, Hasselt, Belgium; Department of Epidemiology and Prevention, Wake Forest School of Medicine, Winston-Salem, USA

**Keywords:** Cardiovascular disease, Cardiovascular risk factor, Survival analysis, Weibull distribution

## Abstract

**Background:**

Epidemiological studies about cardiovascular diseases often rely on methods based on time-to-first-event for data analysis. Without taking into account multiple event-types and the recurrency of a specific cardiovascular event, this approach may underestimate the overall cardiovascular burden of some risk factors, if that is the goal of the study.

**Methods:**

In this study we compare four different statistical approaches, all based on the Weibull distribution family of survival model, in analyzing cardiovascular risk factors. We use data from the Cardiovascular Health Study as illustration. The four models respectively are time-to-first-event only, recurrent-events only, multiple-event-types only, and joint recurrent and multiple-event-type models.

**Results:**

Although the four models produce consistent results regarding the significance of the risk factors, the magnitude of the hazard ratios and their confidence intervals are different. The joint model produces hazard ratios that are substantially higher than the time-to-first-event model especially for the risk factors of smoking and diabetes.

**Conclusion:**

Our findings suggest that for people with diabetes and are currently smoking, the overall cardiovascular burden of these risk factors would be substantially higher than that estimated using time-to-first-event method.

**Electronic supplementary material:**

The online version of this article (doi:10.1186/s12874-015-0004-3) contains supplementary material, which is available to authorized users.

## Background

Smoking, diabetes, blood pressure, and serum cholesterol have long been associated with cardiovascular disease (CVD); they are considered standard risk factors and used in prediction models such as the Framingham Risk Score [1,2]. Risk prediction scores have been derived from prospective cohort studies which typically utilize a time-to-first event/proportional hazards model approach to investigate associations between risk factors and outcomes [3-6]. If multiple events are ascertained, for example a revascularization, myocardial infarction, a stroke, and heart failure, only one is represented in the analysis. However it is this constellation of cardiovascular events, not the one first recorded, that reflects the genuine CVD burden faced by an individual. Adding to the complexity is recurrent events of the same type, if the first event is survived. Recurrent events clearly negatively impact cardiovascular health but are ignored in time-to-first-event analysis.

One naïve way to include both multiple event types and recurrent events is to treat individual events, regardless of event-type and first or recurrent status, as statistically independent. Although this approach circumvents the need for advanced methods and could easily use standard survival analysis techniques, the naïve method produces biases in both the point estimate and the associated standard error. The standard error is deflated because of the apparent increase in the number of observations as a result of including multiple event types and recurring occurrences; this would tend to produce smaller p-values and overly optimistic results. While proper analytic tools that take into account both multiple event-type burdens and recurrency are available, there is little literature about comparing the different methods and their respective findings regarding the assessment of risk factors on overall cardiovascular burden.

Several recurrent models exist in the literature, especially as extensions to the Cox proportional hazard approach. Starting perhaps with a foundational paper by Anderson and Gill, [7], the literature for recurrent data includes the marginal model [8], total time and gap-time models [9,10], random effects/frailty models [11], and a counting process approach [12]. Discussions of the different approaches for recurrent models can be found in other articles [13,14]. Empirical comparison between different recurrent models suggested that results derived from different models could be substantially different, and the optimal model could depend on the context [15]. Models for multiple end-points in survival analysis include the bivariate survival model [16], the marginal model [8], and random effects/frailty models [17]. See Hougaard, [18] for a general overview. Detailed explanation of recurrent and multiple end-points survival models can be found in textbook such as [19].

This study aims to compare traditional time-to-first-event model with approaches that take into account multiple cardiovascular burden and/or recurrency in the context of a prospective cohort study- the Cardiovascular Health Study (CHS). In order to provide direct comparison of the methods and derive a real-world estimate of the extent of understated cardiovascular risk by using time-to-first-event approaches, we selected to use a class of parametric survival models that is amenable to different approaches and then applied the various approaches to a single significant epidemiologic data set – the CHS. The selected parametric survival approaches for recurrent and multiple event-types relied heavily on random effects models. As a result, the direct effects estimated using different approaches cannot be immediately compared. The fixed effect for smoking, in the random effect model, for example, represents an estimate that is *conditional* on the random effect. One way to solve the problem is to “marginalize” the mixed effects models – i.e., first estimate the mixed effects model and then integrate out the random effects based on the estimated parameters from the previous step [20,21]. To summarize, instead of proposing new epidemiologic methods for multiple event-types and recurrent events, our goal here is two-folded: (1) to present results from applying comparable models representing different approaches for handling time-to-event data, and (2) to assess the level of discrepancies in traditional risk reporting on a collection of well-known cardiovascular risk factors of their effects on overall cardiovascular burden.

## Methods

CHS is a prospective population-based cohort study begun in 1989 of risk factors for coronary heart disease (CHD) and stroke in adults 65 years and older; details regarding the sampling strategy and data collection have been published [22,23]. By June 1990, four Field Centers completed the recruitment of 5,201 participants. Between November 1992 and June 1993, an additional 687 African Americans were recruited using similar methods. The Field Centers are located in Forsyth County, NC; Sacramento County, CA; Washington County, MD; and Pittsburgh, PA. The baseline examinations consisted of a home interview and a clinic examination that assessed traditional risk factors. Until 1999, semi-annual contacts alternated between clinic examinations and telephone contacts, during which information about hospitalizations and potential CVD events was collected.

The identification and adjudication of incident events followed a standard protocol. CHS collected hospital records (discharge summaries and appropriate lab/imaging records) for all hospitalizations that were identified by clinic staff via follow-up phone calls and visits. A panel of physician adjudicators then reviewed clinical information for the first of any specific type of events. Initial and subsequent events were identified in the same way and records obtained for all potential events when available, but for subsequent events, because of budget constraints, these records were not sent to the physician panel to verify what the clinics had found. By comparing ICD9 codes from discharge summaries vs. adjudicated endpoint, it was reported that specificity was high (e.g., for myocardial infarction it was 97%), but sensitivity was lower (81%) [21]. Thus, potential endpoints might be missed, but if events were reported via ICD9 it was unlikely that they were misclassified. The adjudicated events are myocardial infarction, angina, heart failure, stroke, transient ischemic attack, coronary revascularization (angioplasty or bypass surgery), claudication, and CHD and non-CHD mortality. In summary, for each event type, only the first event was adjudicated. Events which occurred prior to July 1, 2002 were available for analysis (hence a range of follow-up from 8–13 years for the original and supplemental cohorts).

For all analyses we utilized a de-identified, public-use data set obtained from BIOLINC after IRB approval. The CHS public-use dataset differs from the original dataset in that age is only reported in 2-year bands, and extreme values of continuous variables are truncated to prevent identification of individuals. The use of CHS data was approved by the Institution Review Board of Wake Forest University Health Sciences. The CHS study obtained informed consent from each participant and IRB approvals were obtained from each of the four field sites at Wake Forest University Health Sciences, University of California at Davis, Johns Hopkins University, and the Univeristy of Pittsburg.

### Statistical analysis

The statistical models that we considered include the time-to-first-event survival model, the recurrent event survival model for a single event-type, the multivariate survival model for different event-types, and the multivariate recurrent survival model which takes into account both event-type and recurrent events. For all models, we included as covariates traditional risk factors for CVD (age, systolic blood pressure, use of anti-hypertensive drugs, total and HDL cholesterol, diabetes, smoking - former and current- and family history), as well as body weight categories (normal, overweight, obesity), baseline CVD history, gender, race (White, African American, other) education level, and use of lipid lowering drugs. For direct comparison, all 4 models have similar set ups and are all based on the Weibull hazard model. The Weibull model is a widely used parametric survival model and it posits that the hazard function follows the Weibull distribution, which is characterized by two parameters- the scale parameter *λ*(> 0) and the shape parameter *ρ*(> 0). The scale parameter is reparameterized with regression coefficients. Specifically, the density function of the Weibull distribution can be expressed as follows:

1 $$ f(y)=\lambda \rho {y}^{\rho -1}{e}^{-\lambda {y}^{\rho }}, $$

where *y*(≥0) denote the variable of interest, which in our case is the time to event. When the shape parameter *ρ* = 1, the Weibull model reduces to the exponential model, implying that the hazard is a constant over time. When *ρ* < 1(*ρ* > 1) the hazard decreases (increases) with time. This flexibility is important because the risk for cardiovascular event in CVD patients is unlikely to remain constant over time. Furthermore, the mean and variance from a Weibull model can be directly computed from the two parameters in closed form, making interpretation straightforward. The specifics of the four survival models, which are based on the Weibull distribution in equation (1), are described as follows. In all cases, we assume that participants are statistically independent entities. Additionally, for comparability purpose, we used baseline values of the covariates in all four models.

#### Model 1: Time-to-first-event survival model

The scale parameter in equation (1) is assumed to be a function of a vector of covariates ***x***. To enforce the positivity of the scale parameter, it is standard to employ the exponential function such that

2 $$ \lambda = \exp \left({\beta}_0+{\beta}^T\boldsymbol{x}\right), $$

where *β*_0_ is an intercept term, and *β* is a vector of coefficients. To fully describe the model, we denote the time-to-first-event (any event-type) and covariate features for individual *i* respectively by *y*_*i*_ and ***x***_*i*_. Therefore, Model 1 is specified by the equation:

3 $$ f\left({y}_i\Big|{\boldsymbol{x}}_i\right)=\rho {y_i}^{\rho -1} \exp \left({\beta}_0+{\beta}^T{\boldsymbol{x}}_i\right) \exp \left(- \exp \left({\beta}_0+{\beta}^T{\boldsymbol{x}}_i\right){y_i}^{\rho}\right). $$

#### Model 2: Recurrent-event survival model

This model takes into account the recurrent events but does not distinguish between event types. In order to capture the correlation between recurrent events within the same individual, a common random effect is added to the regression model for observations from the same individual. We denote the time to event *j*, *j* = 1, ⋯, *n*_*i*_, within individual *i*, *i* = 1, ⋯, *N*, by *y*_*ij*_ such that Model 2 is specified by the following equation:

4 $$ f\left({y}_{ij}\Big|{\boldsymbol{x}}_i\right)=\rho {y_{ij}}^{\rho -1} \exp \left({\beta}_0+{\beta}^T{\boldsymbol{x}}_i+{a}_i\right) \exp \left(- \exp \left({\beta}_0+{\beta}^T{\boldsymbol{x}}_i+{a}_i\right){y_{ij}}^{\rho}\right), $$

where *a*_*i*_ denotes the random effect that is common to recurrent events within the same individual. The random effects *a*_*i*_ are assumed to follow a normal distribution of mean 0 and variance *σ*^2^.

#### Model 3. Multivariate event-type survival model

This model is similar to Model 1 except that different event types are distinguished. In other words, a participant who has an event of stroke at day 10, then another event of stroke at day 100 and then an event of angina at day 150 would be counted as encountering two different types of events respectively at day 10 and day 150. We used a different baseline hazard function for each event-type and include a random effect for all event types that an individual experiences. Denoting the time to first event-type *k*, *k* = 1, ⋯, *K*, by *y*_*ik*_, Model 3 is specified by the following equation:

5 $$ f\left({y}_{ik}\Big|{\boldsymbol{x}}_i\right)={\rho}_k{y_{ik}}^{\rho_k-1} \exp \left({\beta}_0+{\beta}^T{\boldsymbol{x}}_i+{b}_i\right) \exp \left(- \exp \left({\beta}_0+{\beta}^T{\boldsymbol{x}}_i+{b}_i\right){y_{ik}}^{\rho_k}\right), $$

where *b*_*i*_ denotes the random effect that is common to different event types the same individual experiences. The baseline hazard, here characterized by the shape parameter *ρ*_*k*_ is assumed to be event-type specific. Similar to Model 2, the random effects *b*_*i*_ are assumed to follow a normal distribution of mean 0 and variance *τ*^2^.

#### Model 4. Joint recurrent and multivariate event-type survival model

This model combines the features in Models 2 and 3 to form the most comprehensive model for capturing CVD burden. We denote the time to recurrent event *j* of event-type *k* within individual *i* by *y*_*ijk*_. Model 4 is characterized by the addition of two random effects:

6 $$ f\left({y}_{ijk}\Big|{\boldsymbol{x}}_i\right)={\rho}_k{y_{ijk}}^{\rho_k-1} \exp \left({\beta}_0+{\beta}^T{\boldsymbol{x}}_i+{c}_{ik}+{d}_i\right) \exp \left(- \exp \left({\beta}_0+{\beta}^T{\boldsymbol{x}}_i+{c}_{ik}+{d}_i\right){y_{ijk}}^{\rho_k}\right), $$

where the event-type random effects *c*_*ik*_ and the individual random effects *d*_*i*_ respectively follow normal distributions of means 0 and respective variances *χ*^2^ and *δ*^2^**.** Additionally, the individual specific random effect is assumed to be independent of the event-type random effect.

In this study, we compared hazard ratio estimates, *e*^*β*^, and 95% confidence intervals for the hazard ratios of relevant risk factors and demographic variables across the 4 models. Because the interpretation of fixed effects within a mixed effects model (i.e., one that includes both fixed and random effects) is different from those estimated from a fixed-effects-only model, we computed estimates from a marginalized model for Model 2 – 4 [19]. This would allow estimates to be fairly compared across the different survival models. The analysis was conducted using SAS PROC NLMIXED (SAS Inc. North Carolina, USA).

## Results

The public-use dataset included 5,795 participants. Table 1 shows descriptive statistics of the sample; of note, approximately one-fifth had pre-existing CVD upon enrollment into CHS. Lipid-lowering drug use was quite uncommon. Over the follow-up period there were 2,920 deaths, corresponding to an annual mortality rate was of 4.9% (95% CI 4.7-5.1). Table 2 shows the number of participants and the respective percentage by event-types. There were a total of 14,349 events in the data set. When considering all events (including CVD-related mortality), approximately 31% of the participants had no events, 27% one event, and 41% had 2 or more events (range 2–44) in the dataset. Note that data from participants with no event over the entire study period or died during the study period were treated as censored observations in all models.

**Table 1:** **Characteristics of the sample. Descriptive statistic – mean (SD) or% of all the predictor variables involved**

	**No.**	**%**	**Mean (SD)**
Gender			
Male	2466	42.6	
Female	3329	57.4	
Race			
White	4855	83.8	
African American	901	15.6	
Others	39	0.67	
Education			
Less than high school	1701	29.5	
High school or above	4071	70.5	
Age group			71-72*
BMI status			
Overweight**	1192	20.6	
Obese	1762	30.4	
Hypertension drug			
Use drug	2749	47.5	
Not use drug	3040	52.5	
Smoking status			
Former smoker	2398	41.4	
Current smoker	698	12.1	
Systolic BP	5784		136.6 (21.9)
Total cholesterol	5739		211.1 (39.3)
HDL	5730		54.2 (15.7)
Diabetes			
Yes	946	16.5	
No	4786	83.5	
Family history of CVD			
Yes	1698	32.0	
No	4492	76.3	
Lipid lowering drug			
Yes	311	5.4	
No	5478	94.6	
Prior CVD			
Yes	1303	22.1	
No	4492	76.3	

*Age is categorized into 13 2-year age groups, starting at age 65 (i.e., ages 65,66 forms the first group), and the last group includes 89 years old and above.

**Overweight is defined as BMI > 27 in males and 25 in females; Obese is defined as BMI > 29.6 in males or 27.3 in females.

**Table 2:** **Statistics of multiple event-types in the CHS cohort**

**Event type**	**Non-fatal**	**Fatal**	**Total**	Percentage*
No event	-	-	1,797	12.5
Myocardial infarction (MI)	961	176	1,137	7.9
Angina	2,872	0	2,872	20.0
Stroke	992	183	1,175	8.2
Congestive heart failure (CHF)	2,964	221	3,185	22.2
Claudication	487	0	487	3.4
Transient ischemic attack (TIA)	337	0	337	2.3
Angioplasty	364	0	364	2.5
Coronary artery bypass	356	0	356	2.5
Electrocardiogram MI (silent)	82	0	82	0.6
CHD-related deaths	0	585	585	4.1
Other deaths	0	1,972	1,972	13.7
**Total**	9,415	3,137	14,349	100

*Percentage is calculated using the total number of events (14,349) as denominator.

**Figure 1:**
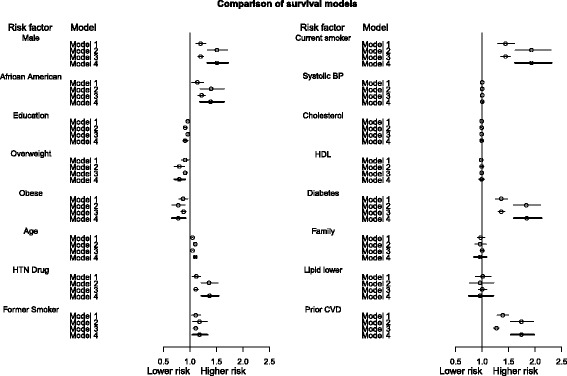
**Summary of hazard ratios and confidence intervals of risk factors.** The summary includes the following survival models: Model 1 (time-to-first-event model, single event type); Model 2 (recurrent event model); Model 3 (multivariate event-type model); Model 4 (multivariate recurrent event model). Circles represent hazard ratios. Confidence intervals for Model 4 are shown as thicker lines.

To summarize the large volume of information generated from the analysis, we used a graph to visualize the results. Figure 1 summarizes the hazard ratio estimates and confidence interval of the risk factors and demographic variables from the 4 models. The specific values of the estimates, confidence limits, and other details about statistical inference are included as Additional file 1, however the following patterns are seen in Figure 1: (1) Models 1 and 3 tend to provide overall lower risk estimates for risk factors compared to other models, (2) Models 2 and 4 provide similar estimates and confidence limits; both have higher risk estimates and wider confidence intervals than Model 1 and 3, (3) Model 3 provides estimates that are similar to Model 1 with slightly shorter confidence intervals, (4) The impacts of using different models are consistent over the various risk factors. For the following risk factors, taking both multiple event-types and recurrent events into account (Model 4) produces noticeably higher hazard ratios than when using the time-to-first-event model (Model 1). The hazard ratio under Model 4 is 1.5 (1.2 under Model 1) for male, 1.39 (1.14) for African American; 1.37 (1.12) for HTN drug; 1.93 (1.44) for current smoker; 1.84 (1.36) for having diabetes; and 1.74 (1.39) for having prior CVD history.

## Discussion

Although comparison between time-to-first-event, recurrent, and multiple-events models have been separately conducted in the literature – a recent example being an sensitivity analysis of recurrence model against time-to-first-event model [24], the current study, as far as we know, is the only study that presents a direct comparison of the 4 models – time-to-first-event, recurrent, multiple events, and joint model for recurrent and multiple events. The analyses presented demonstrate that taking into account multiple event types, as well as recurrent events using appropriate statistical techniques, alters the estimated risk associated with several important risk factors for CVD. The difference is substantial especially for factors such as having diabetes; the hazard ratio when considering both multiple event-types and recurrent events is 36% higher when only considering time-to-first-event. For current smokers, the hazard ratio is 34% higher.

The strengths of this work include the use of a well-characterized prospective cohort study with a long follow-up period, providing a large number of events to analyze, and the application of different statistical approaches under a common class of models to one dataset. One potential limitation which may affect analyses of recurrent events to a greater extent than multiple events types is that subsequent events of the same type were not verified via the adjudication process. Also, use of the public-use dataset may make some risk estimates not directly comparable to prior CHS reports.

There are several implications to our findings. First, time-to-first-event methods tend to substantially underestimate overall risk of important CVD burden of factors such as smoking and diabetes. Using time-to-first-event methods also tends to underestimate disparities across demographic background. The CVD burden for African Americans, for example, is 21% higher (in hazard ratio) than Whites when both multiple event types and recurrences are taken into account. This study also demonstrates that in long term studies such as the CHS, multiple event types and recurrent events are not rare at all. Indeed, in the CHS cohort, 41% had 2 or more events over the 8–13 years of follow-up. The substantial proportion of participants that suffered from recurrent and different cardiovascular event-types is simply too large to be ignored.

We were surprised by the direction of the hazard ratio for obesity in all four models. It has been documented in the literature that an overweight body mass index (BMI) is associated with lower all-cause mortality or cardiovascular mortality risks in the elderly but not younger adults [25]. The so-called obesity paradox could perhaps partially explain the seemingly protective effect of overweight/obese, as suggested by the current analysis. In order to further verify this finding, we conducted an exploratory analysis using visualization and descriptive statistics. The exploratory analysis suggested that BMI almost have no or little effect on the risk of a cardiovascular event in this population. A summary graph of the analysis is provided as Additional file 1.

Our findings also suggest that among the two additional considerations – multiple event-types and recurrent events – the argument for including the recurrent component in the model is stronger. When a recurrent model - such as Model 2 in the current paper - takes into account all recurrent events, it will capture a substantial portion of the variance that arises from multiple occurrences of cardiovascular event, even when the types of events are not distinguished. It also needs to be pointed out that different models could serve different purposes. The comprehensive model (Model 4) aims to examine long-term and overall CVD burden while Model 1 is suitable for assessing risk of the first cardiovascular event of a specific type. In summary, the recurrent and multiple event-type methods described in this paper may be particularly well suited towards understanding the burden of illness across populations using claims data or other electronic health record systems, where surveillance for multiple event types and recurrent events may not impose significantly increased study costs. These methods may also be useful for cohort studies or clinical trials where the outcomes may frequently be repeated (e.g. heart failure hospitalization).

The study has limitations. First, we directly apply four different models to a real data set and assess the discrepancies between the risk estimates. However, we do not know the “true” model. Thus a limitation of the study is that there is no simulation experiment to evaluate the validity of the models. However, it is important to point out the limitation of simulation experiments in such an emulative study - the optimally fitted model would depend on the choice of the generative model. For example, if Model 4 is used as the generative or true model, then misspecified models (Models 1–3) are expected to provide biased estimates. The same can be said about using other models as generative models. The value of using a significant real data set is that the analysis could uncover the magnitude and direction of potential bias if the “ground truth” is that both recurrent events and multiple event types do contribute to the overall cardiovascular risk burden of patients.

We contend that other than Model 4, alternative and more refined models could be developed for multivariate recurrent survival data. For example, existing multilevel models [26] can be extended to provide event-specific parameter estimates of risk factors. In this paper we do not distinguish between potentially different effects of risk factors on different event types as our interest is in assessing overall cardiovascular burden. In a way, the overall cardiovascular burden analysis is analogous to analyzing sum scores of symptoms using a symptom checklist as opposed to analyzing each symptom individually. The two approaches serve different purposes and are both useful for answering different questions.

A second limitation is the use of a parametric family of distribution, namely the Weibull family, for modeling time-to-event. While the Weibull distribution is well known for its flexibility for incorporating increasing, decreasing, and constant hazard rates, there are other models such as the semi-parametric Cox proportional hazard model that are equally flexible, if not more. The main reason we selected the Weibull family for all models is that we can have a computationally tractable and common statistical framework for direct comparison across multiple models. Finally, we need to caution that the reported results are based on a single, albeit large-scale epidemiologic study, and the findings may not be always generalizable.

## Conclusion

This comparison study of several different time-to-event models demonstrates that the model that takes into account both recurrent events and multiple event types better reflects the overall cardiovascular burden of adults in the US. For people with diabetes and are currently smoking, the overall cardiovascular burden of these risk factors would be substantially higher than that estimated using time-to-first-event method.

## Additional file

Additional file 1:
**Model parameter estimates and 95% confidence interval; graph showing distribution of BMI and number of events with fitted smoothed curve.**

## Abbreviations

CVD: Cardiovascular disease
CHS: Cardiovascular health study
CHD: Coronary heart disease
BMI: Body mass index
