# Supplementary material for: Comparison of risks of cardiovascular events in the elderly using standard survival analysis and multiple-events and recurrent-events methods
Source: BMC Med Res Methodol. 2015 Mar 8;15:15. doi: 10.1186/s12874-015-0004-3 (PMC4364095; doi:10.1186/s12874-015-0004-3)
Supplement: Additional file 1: — Model parameter estimates and 95% confidence interval; graph showing distribution of BMI and number of events with fitted smoothed curve. [file 12874_2015_4_MOESM1_ESM.pdf]

Additional Table: Estimates of hazard ratios for four respective models and 95% confidence limits.

	Model 1			Model 2			Model 3			Model 4		
Effect	Est	LL	UL	Est	LL	UL	Est	LL	UL	Est	LL	UL
Hypertension drug	1.12	1.04	1.20	1.36	1.21	1.53	1.11	1.07	1.16	1.37	1.22	1.54
Male	1.20	1.11	1.30	1.51	1.33	1.71	1.20	1.15	1.26	1.51	1.33	1.72
Age	1.05	1.04	1.06	1.10	1.08	1.11	1.05	1.05	1.06	1.10	1.09	1.11
BMI2	0.91	0.84	0.98	0.80	0.70	0.90	0.91	0.87	0.95	0.80	0.71	0.91
BMI3	0.87	0.79	0.96	0.78	0.66	0.91	0.88	0.83	0.93	0.78	0.66	0.92
Afr.Am race	1.14	1.03	1.26	1.40	1.19	1.65	1.22	1.15	1.29	1.39	1.19	1.64
Former smoker	1.11	1.03	1.20	1.18	1.05	1.33	1.11	1.06	1.15	1.18	1.05	1.33
Current smoker	1.44	1.29	1.61	1.93	1.62	2.30	1.44	1.35	1.53	1.93	1.62	2.31
SBP	1.00	1.00	1.00	1.00	1.00	1.01	1.00	1.00	1.00	1.00	1.00	1.01
Cholesterol	0.99	0.98	1.00	0.99	0.97	1.00	0.99	0.99	1.00	0.99	0.97	1.00
HDL	0.98	0.96	1.01	0.99	0.95	1.03	0.99	0.97	1.00	0.99	0.95	1.03
Diabetes	1.36	1.25	1.48	1.83	1.59	2.10	1.36	1.30	1.43	1.84	1.60	2.12
Family CVD history	0.97	0.91	1.05	0.96	0.86	1.08	1.00	0.96	1.04	0.96	0.85	1.08
Education	0.96	0.93	0.98	0.91	0.88	0.95	0.96	0.95	0.98	0.91	0.88	0.95
Lipid Lowering Drug	1.01	0.87	1.17	0.96	0.76	1.22	1.00	0.93	1.09	0.96	0.75	1.21
Prior CVD	1.39	1.28	1.50	1.74	1.53	1.97	1.27	1.21	1.32	1.74	1.54	1.98

Est = Estimate; LL= Lower limit of 95% confidence interval; UL=Upper limit of 95% confidence interval; BMI2= overweight (BMI 25-29.9) , BMI 3=obese (BMI$\geq$30)

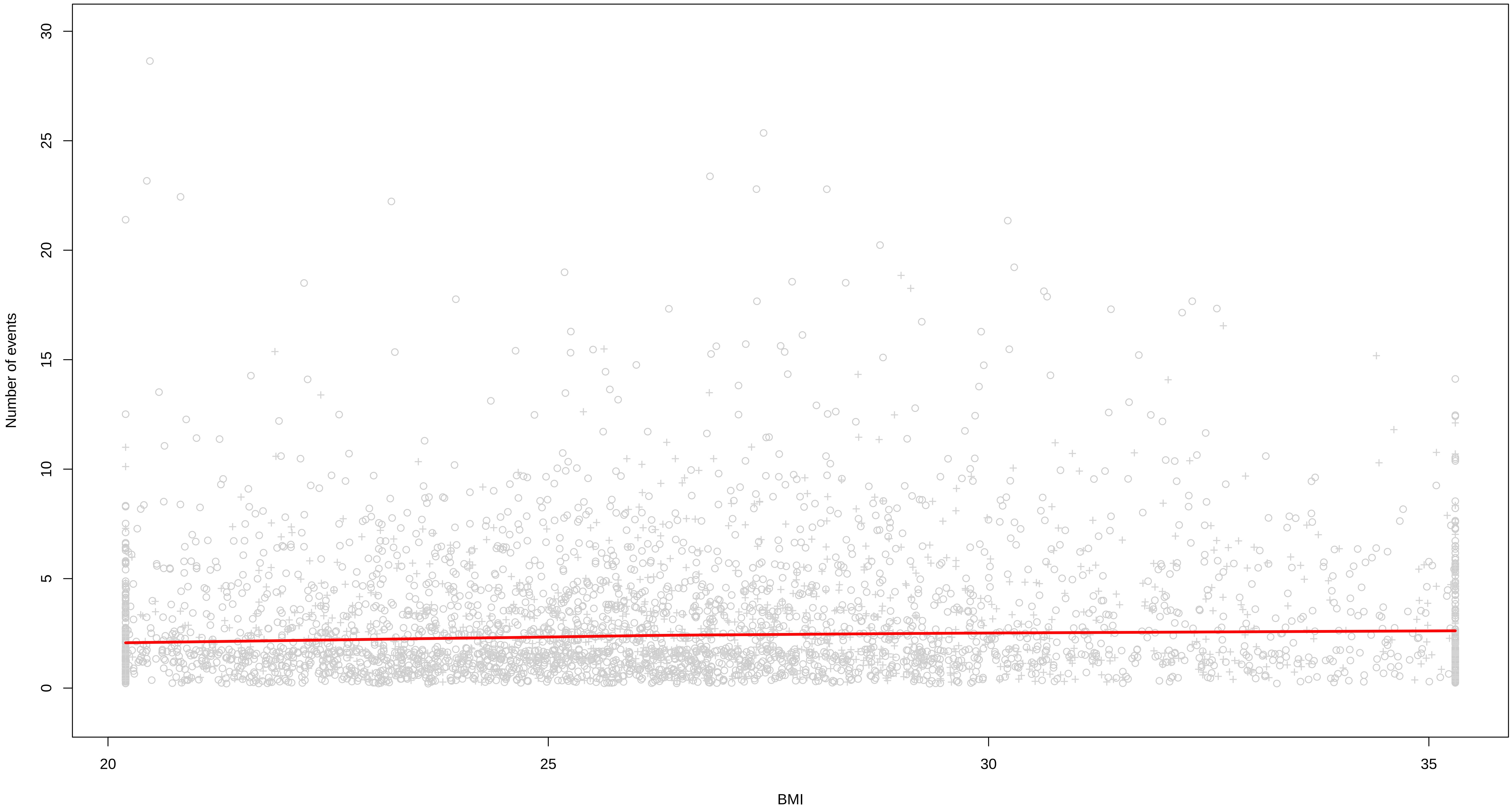

Number of events
BMI
0
5
10
15
20
25
30
20
25
30
35
